# Supplementary material for: A truncating mutation in the autophagy gene UVRAG drives inflammation and tumorigenesis in mice
Source: Nat Commun. 2019 Dec 12;10:5681. doi: 10.1038/s41467-019-13475-w (PMC6908726; doi:10.1038/s41467-019-13475-w)
Supplement: Supplementary file 1 — Supplementary Information [file 41467_2019_13475_MOESM1_ESM.pdf]

Supplementary Information

A truncating mutation in the autophagy gene UVRAG drives inflammation and tumorigenesis in mice

Quach, Song, and Guo et al.

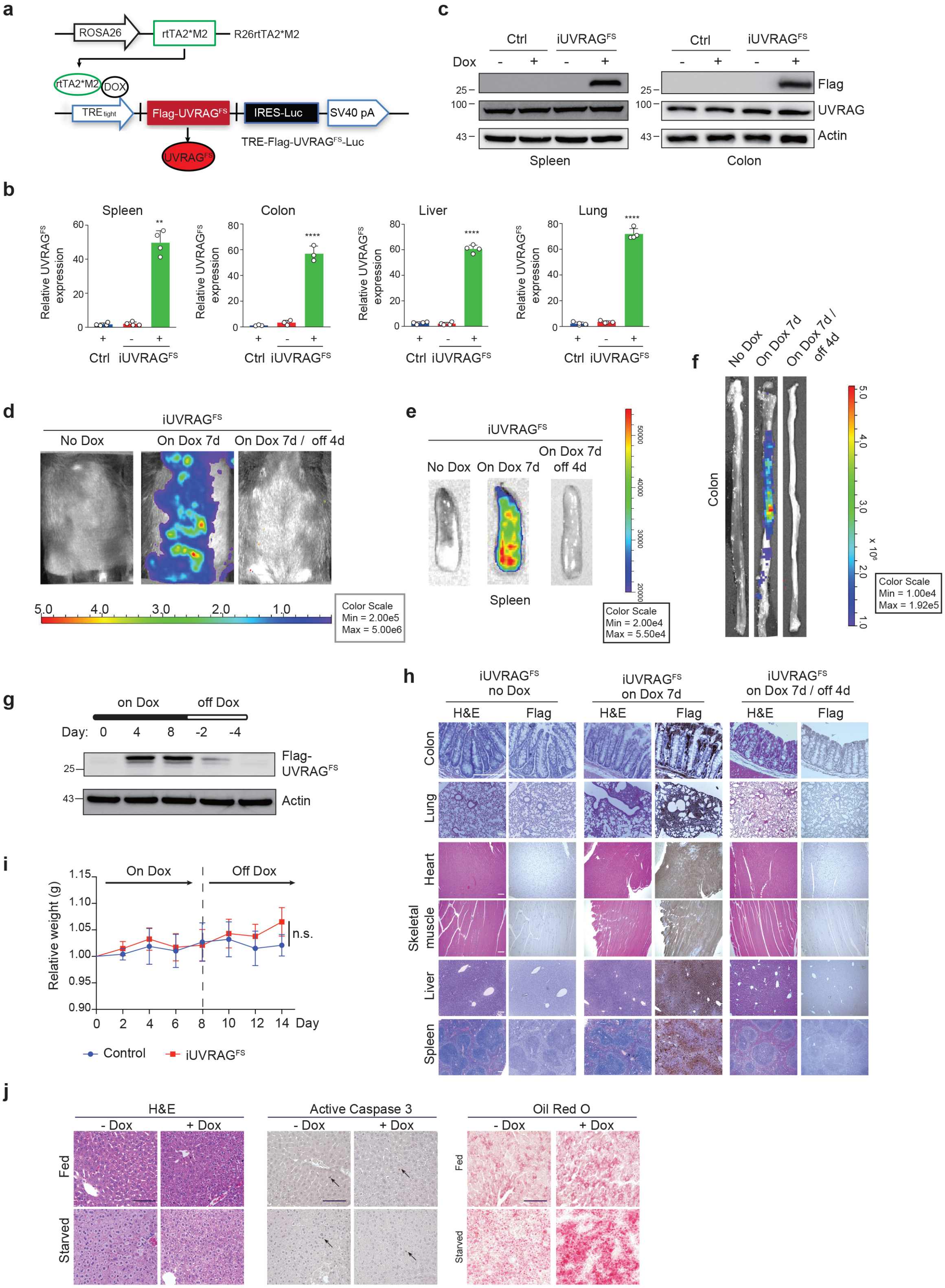

Supplementary Figure 1

Supplementary Figure 1. Dox-inducible expression of UVRAG$^{\mathbf{FS}}$ in mice.
a Schematic diagram of the system used for Dox-inducible expression of *UVRAG^{FS}*. Dox, doxycycline. Luc, luciferase.
b Levels of *UVRAG^{FS}* transcript were evaluated by quantitative RT-PCR in different organs from mice of indicated genotype (n = 3-4 mice per genotype).
c Western blot (WB) analysis of transgenic Flag-UVRAG^{FS} and endogenous UVRAG expression in spleens and colons from mice of indicated genotype with or without Dox treatment for 7 days.
d *in vivo* bioluminescent imaging of the abdominal region of representative *iUVRAG^{FS}* mice treated with Dox as indicated.
e,f Bioluminescent imaging of the spleen (**e**) and colon (**f**) from *iUVRAG^{FS}* mice treated with Dox as indicated.
g WB analysis of UVRAG^{FS} expression in the spleens from *iUVRAG^{FS}* mice treated with Dox as indicated. Actin serves as a loading control.
h Representative H&E (left panel) and Flag immunohistochemical (IHC) (right panel) sections of the colon, lung, heart, skeletal muscle, liver, and spleen from *iUVRAG^{FS}* mice treated with Dox as indicated.
i Relative weight of control and *iUVRAG^{FS}* mice with and without Dox treatment over 14 days. n.s., not significant.
j Representative H&E-stained section (left panel), immunohistochemical (IHC) staining of cleaved caspase 3 (middle panel), and Oil red O staining (right panel) of the livers from fed and 48-h-starved *iUVRAG^{FS}* mice with or without Dox treatment. Arrows indicate apoptotic cells.

Scale bars, 100 µm. Data in (**c, g**) are from one experiment that is representative of three independent experiments. Data in (**d-f, h, j**) are from one animal that is representative of 5-12 animals in each group. For all quantifications, data (mean ± SD) were from the indicated number of independent experiments and analyzed with one-way ANOVA using Graphpad Prism 7.0 software. Source data are provided as a Source Data file. n.s., not significant; **, $P < 0.01$; ****, $P < 0.0001$. See Supplementary Fig. 9 for uncropped data of **c, g.**

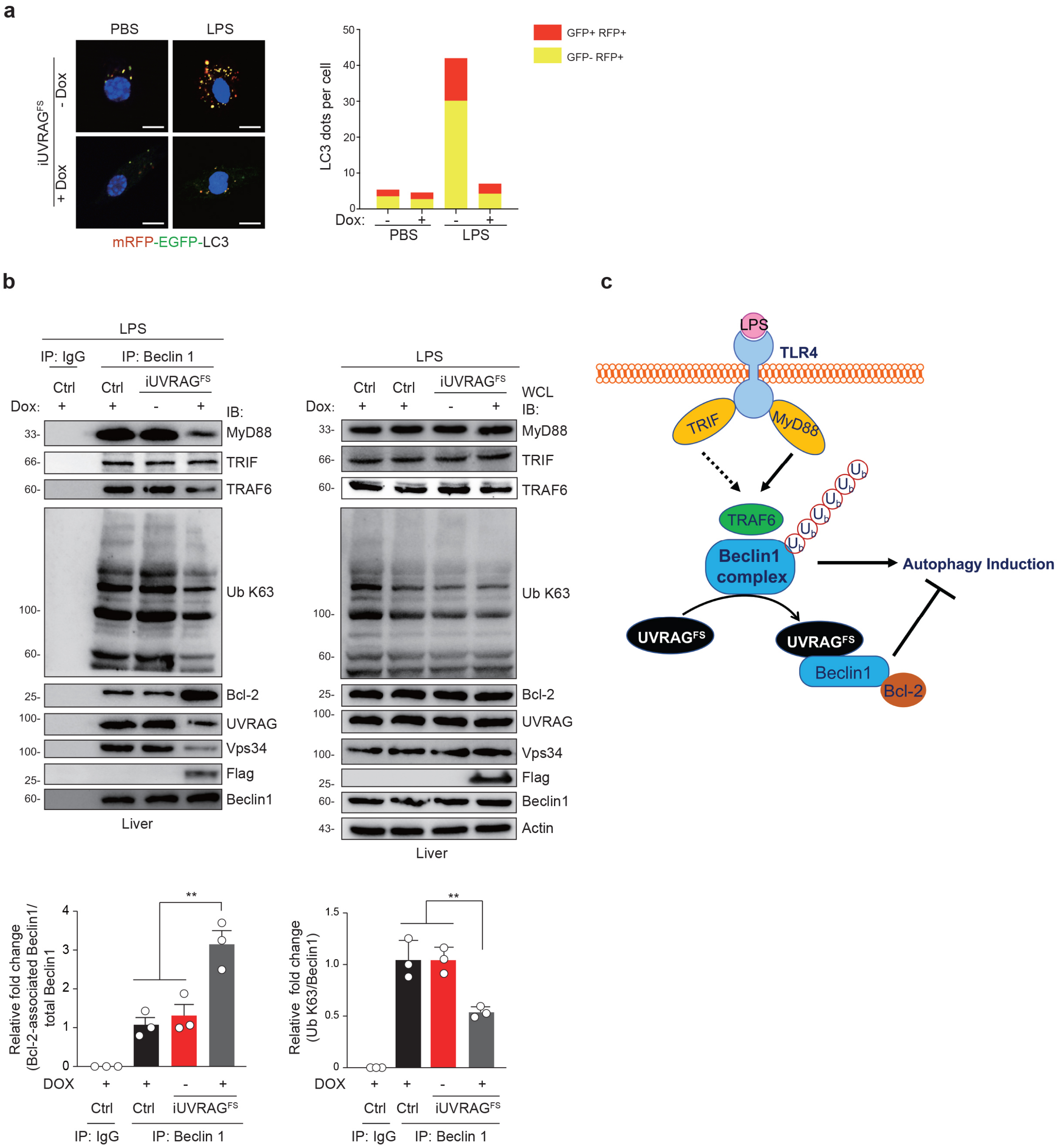

Supplementary Figure 2

Supplementary Figure 2. Effects of $UVRAG^{FS}$ on LPS-induced autophagy activation.
a $UVRAG^{FS}$ expression inhibits LPS-induced autophagic response. *iUVRAG*^{FS} BMDM transfected with mRFP-EGFP-LC3 were treated with PBS or LPS (100 ng/ml) for 4 h in the presence/absence of Dox. Representative confocal images of mRFP-EGFP-LC3 puncta in cells were shown (left) and the numbers of $GFP^{+}RFP^{+}$ puncta (yellow dots; neutral autophagosome) and $GFP^{-}RFP^{+}$ puncta (red dots; matured autophagosome) per cell were quantified (right). Scale bars, 10 µm. n = 50-100 cells pooled from three independent experiments.
b Co-immunoprecipitation (co-IP) of indicated proteins with Beclin1 in the livers from control and *iUVRAG*^{FS} mice, treated with Dox and LPS as indicated. Expression of indicated proteins in WCLs are shown (right panel). Densitometric quantification of the Bcl-2-associated Beclin1/total Beclin1 and the ubiquitinated (K63)-Beclin1/total Beclin1 ratios in the livers of the indicated genotypes are also shown (bottom panels).
c Schematic diagram showing the mechanism by which $UVRAG^{FS}$ disrupts the LPS-induced Beclin1 recruitment to TLR4 receptor complex and enhances its interaction with negative regulator Bcl-2 by regulating TRAF6-mediated K63 ubiquitination of Beclin1.

Data in (**b**) are from one experiment that is representative of three independent experiments. For all quantifications, data (mean ± SD) were from the indicated number of independent experiments and analyzed with one-way ANOVA using Graphpad Prism 7.0 software. Source data are provided as a Source Data file. n.s., not significant; **, $P < 0.01$. See Supplementary Fig. 9 for uncropped data of **b.**

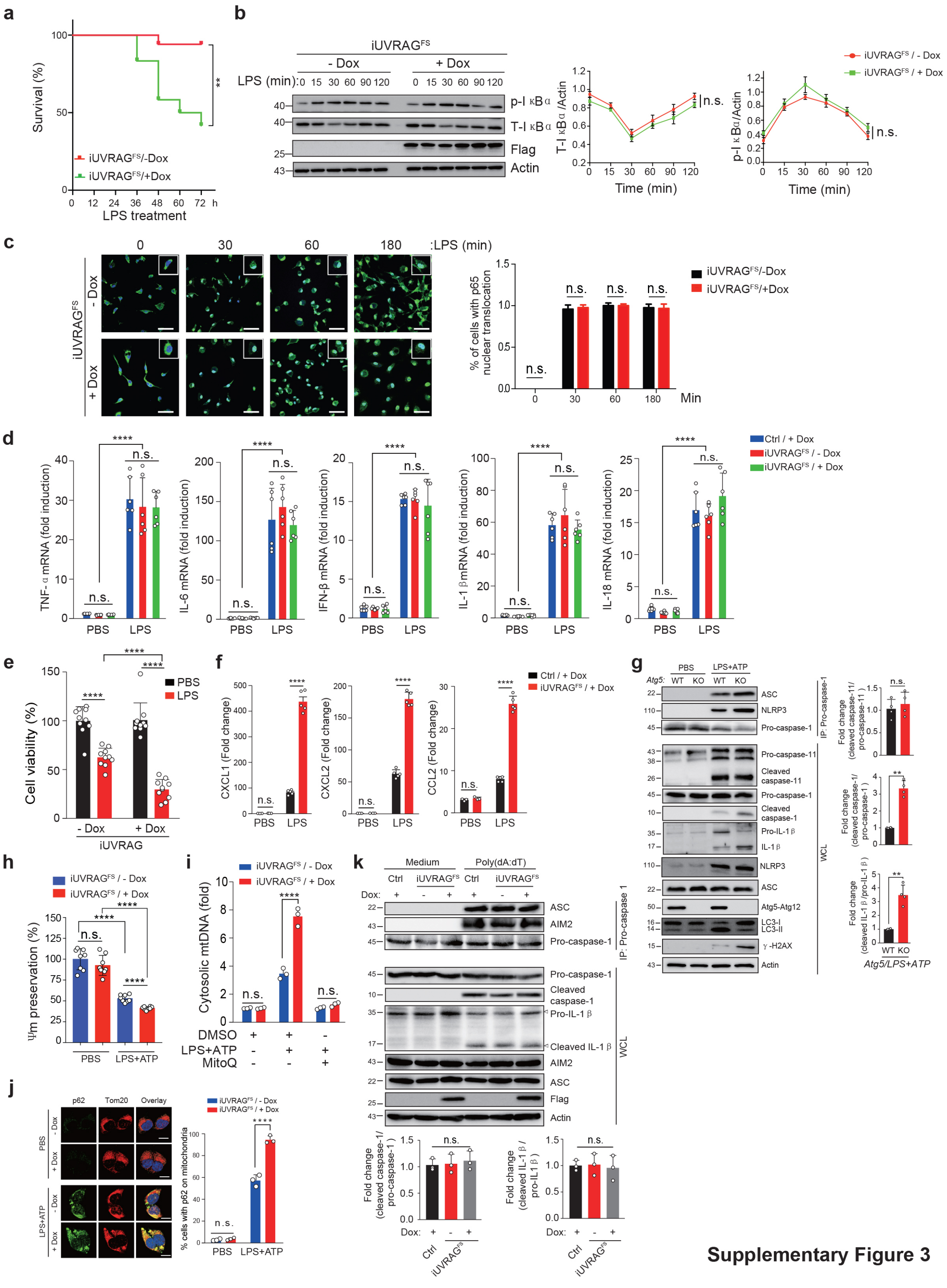

Supplementary Figure 3

Supplementary Figure 3. UVRAG^{FS} enhances septic shock by promotion of inflammatory responses.
a Kaplan-Meier curve showing survival of Dox-treated/untreated *iUVRAG^{FS}* mice (n = 10 per group) challenged with LPS (20 mg per kg body weight; *i.p.*). **, $P < 0.01$ (Log-rank test).
b WB (left) and densitometric quantification (right) of phosphorylated (p-)IκBα and total IκBα in *iUVRAG^{FS}* BMDMs stimulated with LPS.
c Immunofluorescence analysis (left) and quantification (right) of relative distribution of endogenous p65 (green) in the nucleus of cells in (**b**). Scale bars, 50 μm.
d Quantitative RT-PCR analysis of indicated cytokine genes in spleen of *iUVRAG^{FS}* mice and littermate control challenged with LPS. n = 6 mice per genotype per condition.
e Cell viability of *iUVRAG^{FS}* BMDMs stimulated with PBS or LPS. n = 10.
f Relative chemokines mRNA expression as indicated by quantitative RT-PCR in spleens from LPS-challenged mice. n = 3-5 mice per genotype per condition.
g Co-IP of ASC and NLRP3 with pro-caspase-1 and WB analysis of indicated proteins in BMDM from Atg5 wild-type and knockout mice, treated with PBS or LPS+ATP. Densitometric quantification of production of cleaved caspase-1 and IL-1β to their precursors in cell culture is shown (right).
h Relative mitochondrial membrane potential (Δψm) changes in LPS+ATP-stimulated *iUVRAG^{FS}* BMDM. n = 8.
i Relative mtDNA concentrations in LPS+ATP-treated *iUVRAG^{FS}* BMDM in the presence/absence of MitoQ treatment. n =3.
j Immunofluorescence (left) and quantification (right) of p62 distribution (green) relative to Tom20-labelled mitochondria (red) in LPS+ATP-stimulated *iUVRAG^{FS}* BMDM. Scale bars, 10 μm.
k Co-IP of ASC and AIM2 with pro-caspase-1 and WB of indicated proteins in WCLs of BMDM from *iUVRAG^{FS}* and control mice transfected with vehicle or Poly(dA:dT). Densitometric quantification of relative production of cleaved caspase-1 and IL-1β to their precursors in BMDM culture is shown (bottom).

Data in (**c, j**) are from one animal representative of 10 animals in each group. For all quantifications, data (mean ± SD) were from the indicated number of independent experiments and analyzed with Student's *t*-test (**g, h**), one-way (**k**) and two-way ANOVA (**b, d-f, i-j**) using Graphpad 7.0 Prism software. Source data are provided as a Source Data file. n.s., not significant; **, $P < 0.01$; ***, $P < 0.001$; ****, $P < 0.0001$. See Supplementary Fig. 9 for uncropped data of **b, g, k.**

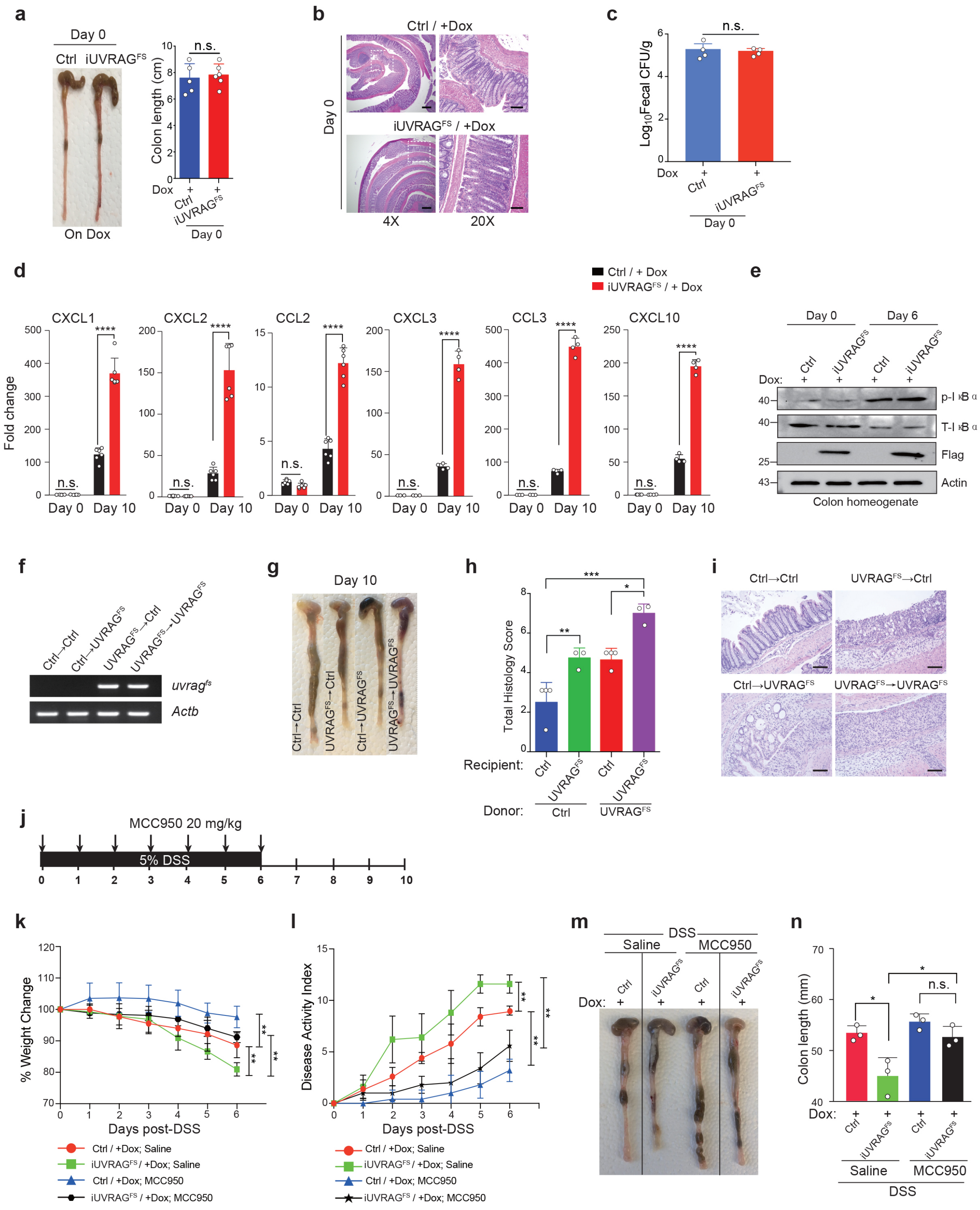

Supplementary Figure 4

Supplementary Figure 4. UVRAG^{FS} enhances inflammatory response in DSS-colitis.
a Representative macroscopic images (left) and length of colon (right) from mice of indicated genotypes at Day 0 in colitis model.
b H&E sections of the colons from mice in (**a**). Scale bars, 300 μm (left) and 100 μm (right).
c The number of bacteria in feces of control and UVRAG^{FS}-expressing mice. CFU, colony forming unit. n = 4 mice per genotype.
d Relative mRNA expression of the neutrophil chemokines CXCL1, CXCL2, CXCL3, CCL2, CCL3, and CXCL10 as determined by quantitative RT-PCR in the colons from mice of indicated genotypes. n = 4-6 mice.
e WB analysis of phosphorylated (p-)IκBα and total IκBα in the colon from Dox-treated mice of indicated genotype on day 0 and day 6 of DSS treatment.
f RT-PCR analysis for UVRAG^{FS} reconstitution in peripheral leukocytes in 12-week-old *UVRAG^{FS}* bone marrow-chimeric mice. Control → control, control mice reconstituted with control BM-derived cells; control → UVRAG^{FS}, UVRAG^{FS}-expressing mice reconstituted with control BM-derived cells; UVRAG^{FS} → control, control mice reconstituted with UVRAG^{FS}-expressing BM-derived cells; UVRAG^{FS} → UVRAG^{FS}, UVRAG^{FS}-expressing mice reconstituted with UVRAG^{FS}-expressing BM-derived cells.
g Representative macroscopic images of the colons from DSS-treated *UVRAG^{FS}* BM chimera on day 10.
h, i Histopathology scores (**h**) and H&E sections (**i**) of the colons from *UVRAG^{FS}* chimeric mice as indicated in DSS-induced colitis. n = 3 mice per group. Scale bars, 100 μm.
j Schematic representation of the experimental procedure for targeting NLRP3 inflammasome in DSS-induced colitis. Dox-treated control and *iUVRAG^{FS}* mice were treated with 5% DSS for 6 days, followed by 4 days of regular water. These mice were injected daily from days 0 to 6 with MCC950 (20 mg per kg body weight) or vehicle control.
k, l Relative weight change (**k**) and DAI (**l**) of Dox-treated control and *iUVRAG^{FS}* mice in (**j**) in response to MCC950 therapy in DSS-induced colitis.
m, n Representative macroscopic images (**m**) and length of colon (**n**) from MCC950-treated mice of indicated genotypes (n = 3 mice) during DSS-induced colitis.

Data in (**e**) are from one experiment that is representative of three independent experiments. Data in (**a, b, f-i, m**) are from one animal that is representative of 3-10 animals in each group. For all quantifications, data (mean ± SD) were from the indicated number of independent experiments and analyzed with Mann Whitney U (**a**), Student's *t*-test (**c**), one-way ANOVA (**h**) and two-way ANOVA (**d, n**) using Graphpad Prism 7.0 software. Source data are provided as a Source Data file. n.s., not significant; *, $P < 0.05$; **, $P < 0.01$; ***, $P < 0.001$; ****, $P < 0.0001$. See Supplementary Fig. 9 for uncropped data of **e.**

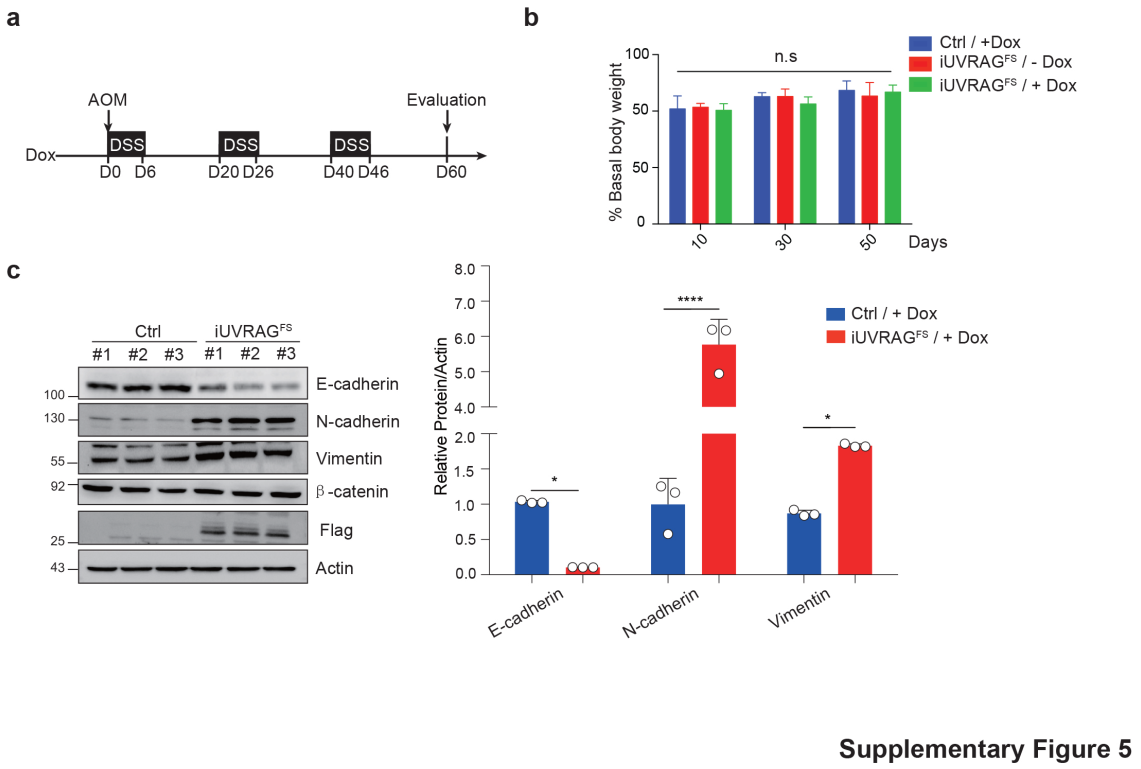

Supplementary Figure 5. UVRAG^{FS} expression inhibits cell differentiation in AOM-DSS model.
a Schematic representation of the experimental procedure for the AOM-DSS model. Mice were injected with AOM (10 mg per kg body weight) on day 0, followed by 3 cycles of 2.5% DSS given in the drinking water (black boxes) for 6 days separated by 2 weeks of regular water. Mice were euthanized on day 60.
b Weight change during AOM-DSS treatment in mice of indicated genotypes was expressed as the percentage of change from day 0. n = 5-12 mice per genotype.
c WB analyses (left) and densitometric quantitation (right) of E-cadherin, N-cadherin, Vimentin, and β-catenin in the colons from Dox-treated mice of indicated genotypes during AOM-DSS treatment (three randomly chosen samples per group; similar results observed in all 5-12 samples per group).

For all quantifications, data (mean ± SD) were from the indicated number of independent experiments and analyzed with two-way ANOVA using Graphpad Prism 7.0 software. Source data are provided as a Source Data file. n.s., not significant; *, $P < 0.05$; ****, $P < 0.0001$. See Supplementary Fig. 9 for uncropped data of **c.**

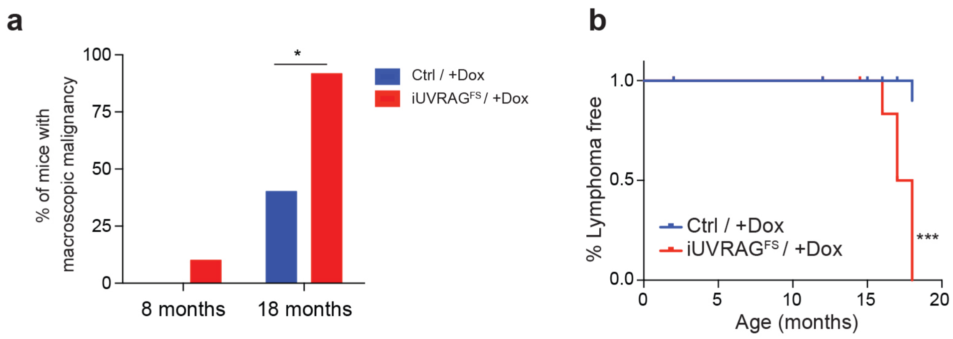

Supplementary Figure 6

Supplementary Figure 6. UVRAG^{FS} expression promotes spontaneous tumorigenesis.

a Prevalence of macroscopic malignancies in 8-month-old (n = 10) and 18-month-old (n = 23) Dox-treated *iUVRAG*^{FS} mice versus Dox-treated control mice. $P < 0.05$ (Fisher's exact test). *, $P < 0.05$ (Fisher's exact test).

b Kaplan-Meier plot of time to development of lymphoma in Dox-treated *iUVRAG*^{FS} mice (n = 19) versus Dox-treated control mice (n = 24 mice per group). (***, $P < 0.0001$; Log-rank test). Lymphoma is determined by complete histologic survey of all major internal organs.

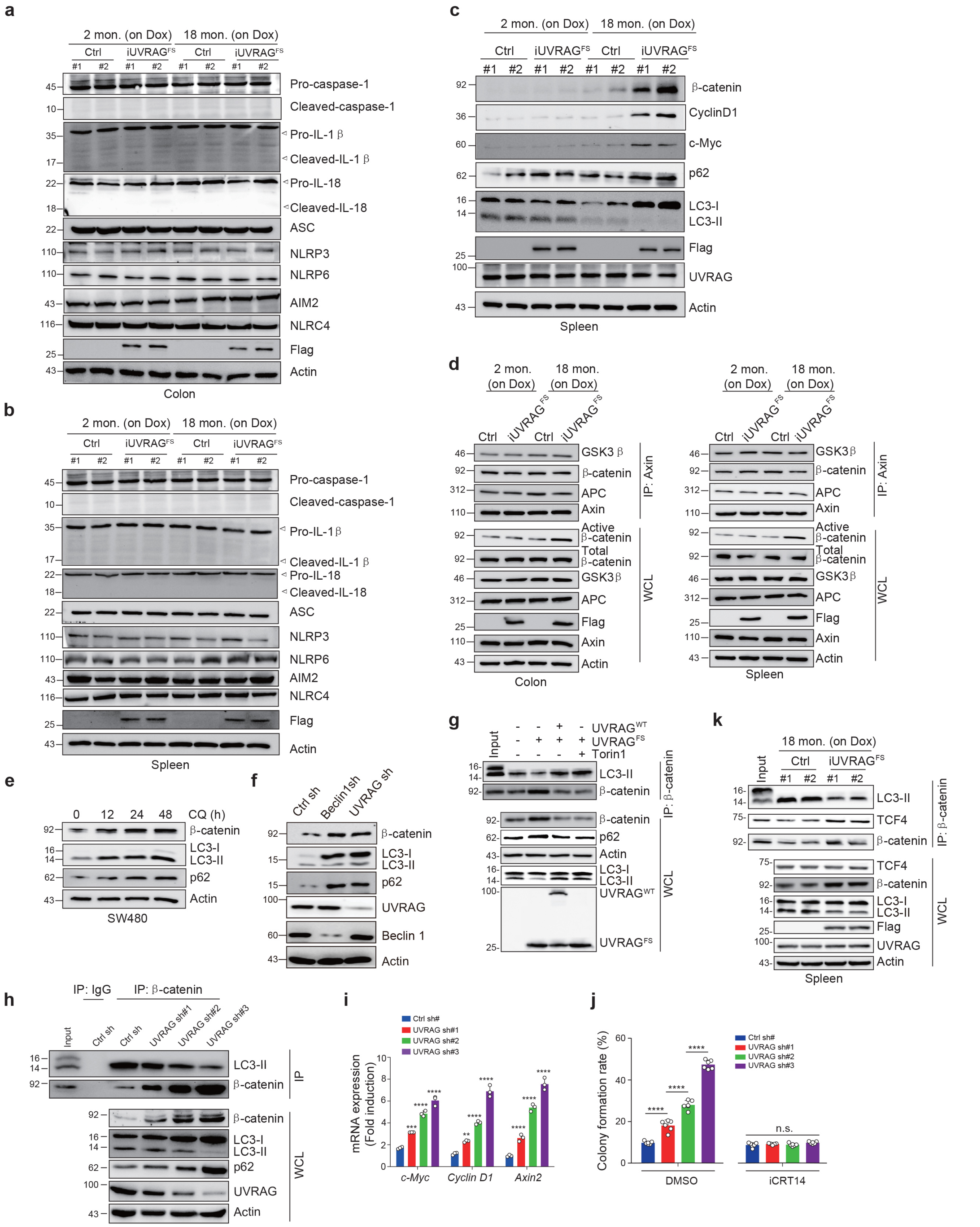

Supplementary Figure 7

Supplementary Figure 7. UVRAG^{FS} promotes cell proliferation by β-catenin activation.
a,b WB of caspase-1 cleavage, IL-1β and IL-18 production, ASC, NLRP3, NLRP6, AIM2, NLRC4 in the colon (**a**) and the spleen (**b**) from 2-month-old and 18-month-old Dox-treated control and *iUVRAG^{FS}* mice (two randomly chosen samples per group; similar results observed in all 10 samples per group).
c WB analysis of indicated proteins in the spleen from 2-month-old and 18-month-old Dox-treated control and *iUVRAG^{FS}* mice (two randomly chosen samples per group; similar results observed in all 10 samples per group).
d Co-IP of GSK3β, APC, and β-catenin with Axin1 in the colons (left) and spleens (right) from 2-month-old and 18-month-old Dox-treated control and *iUVRAG^{FS}* mice.
e WB analysis of β-catenin and autophagy markers in SW480 cells treated with 3-MA (1 mM) or with CQ (20 μM) for the indicated time. Actin serves as a loading control.
f Knockdown of autophagy proteins stabilizes β-catenin. SW480 cells were transfected with control shRNA, Beclin1- or UVRAG-specific shRNA, followed by WB analysis of indicated proteins.
g Wild-type UVRAG and autophagy induction antagonizes the effect of UVRAG^{FS} on β-catenin stabilization. SW480 cells stably expressing UVRAG^{FS} were treated with Torin 1 (1 μM, 6 h) or transfected with wild-type UVRAG, followed by IP with β-catenin and IB with indicated antibodies.
h UVRAG knockdown suppresses autophagy and stabilizes β-catenin. SW480 cells were transfected with three different UVRAG shRNA. WCL were used for co-IP with anti-β-catenin, followed by IB with the indicated antibodies. Note that β-catenin increase correlates with shRNA knockdown efficiency.
i Quantitative RT-PCR analysis of indicated gene expression in cells in (**h**). n = 3.
j Colony formation assay of SW480 cells stably expressing control shRNA or UVRAG shRNA in (**h**), after treatment with DMSO or iCRT14 (50 μM, 14 days). n =6.
k Co-IP of β-catenin with LC3 and TCF4 in the spleens from 18-month-old mice of indicated genotype (two randomly chosen samples per group; similar results observed in all 10 samples per genotype).

Data in (**d-h**) are from one experiment that is representative of three independent experiments. For all quantifications, data (mean ± SD) were from the indicated number of independent experiments and analyzed with two-way ANOVA using Graphpad Prism 7.0 software. Source data are provided as a Source Data file. n.s., not significant; **, $P < 0.01$; ***, $P < 0.001$; ****, $P < 0.0001$. See Supplementary Fig. 9 for uncropped data of **a-h, k.**

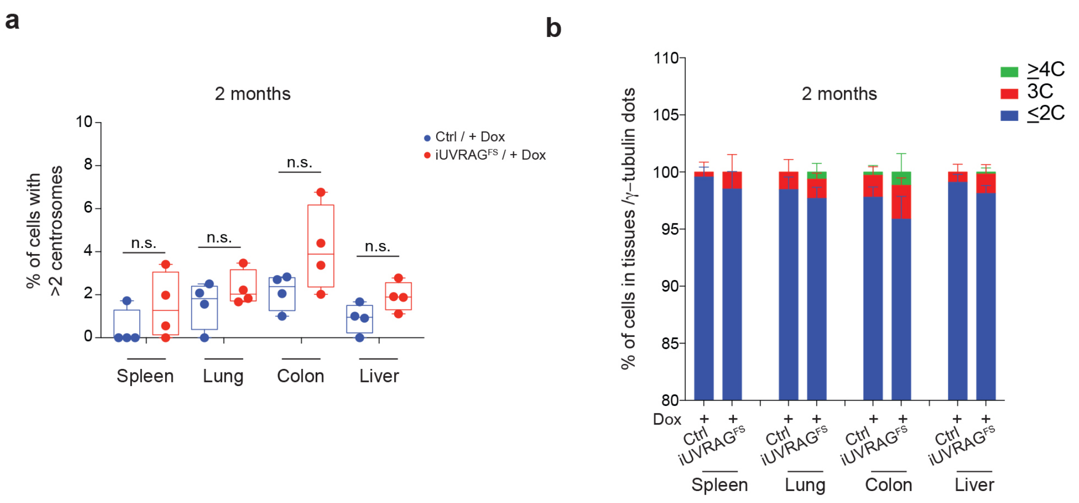

Supplementary Fig. 8

Supplementary Figure 8. Centrosome amplification associated with UVRAG^{FS} expression in vivo.
a Quantification of the level of centrosome amplification in indicated tissues from Dox-treated 2-month-old control (n = 4) and *iUVRAG^{FS}* mice (n = 4).
b Quantification of centrosome numbers in indicated tissues from 2-month-old Dox-treated control (n = 4) and *iUVRAG^{FS}* mice (n = 4). C, centrosomes.

Data represents the mean ± SD derived from the indicated number of independent experiments. Source data are provided as a Source Data file. n.s., not significant (Student's *t* test).

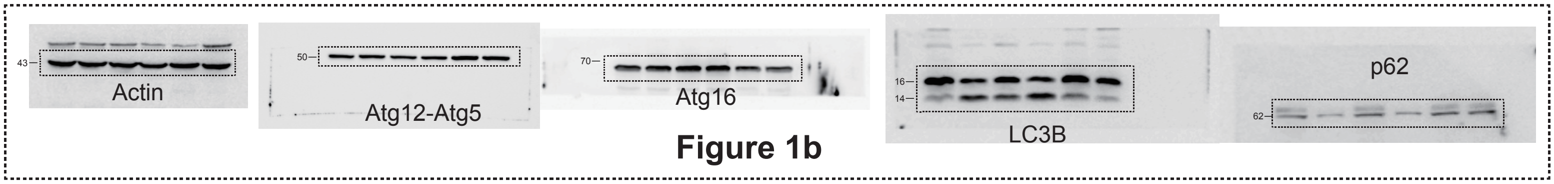

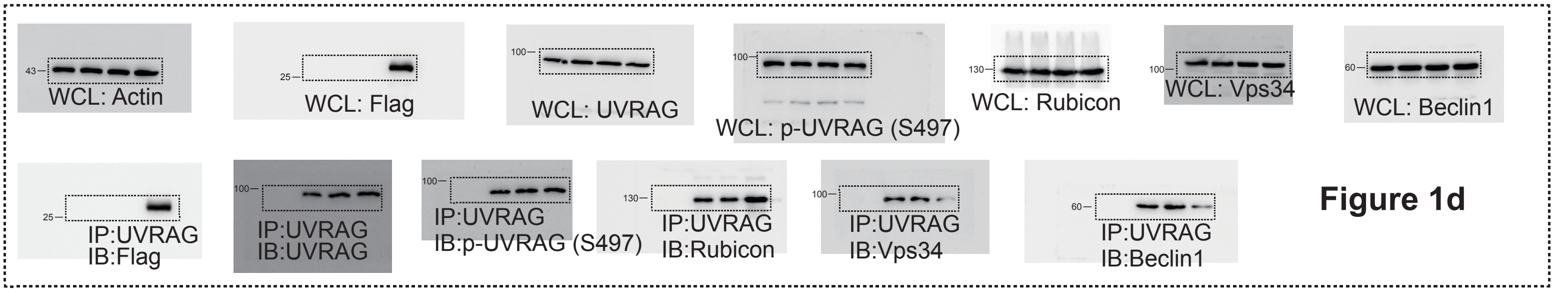

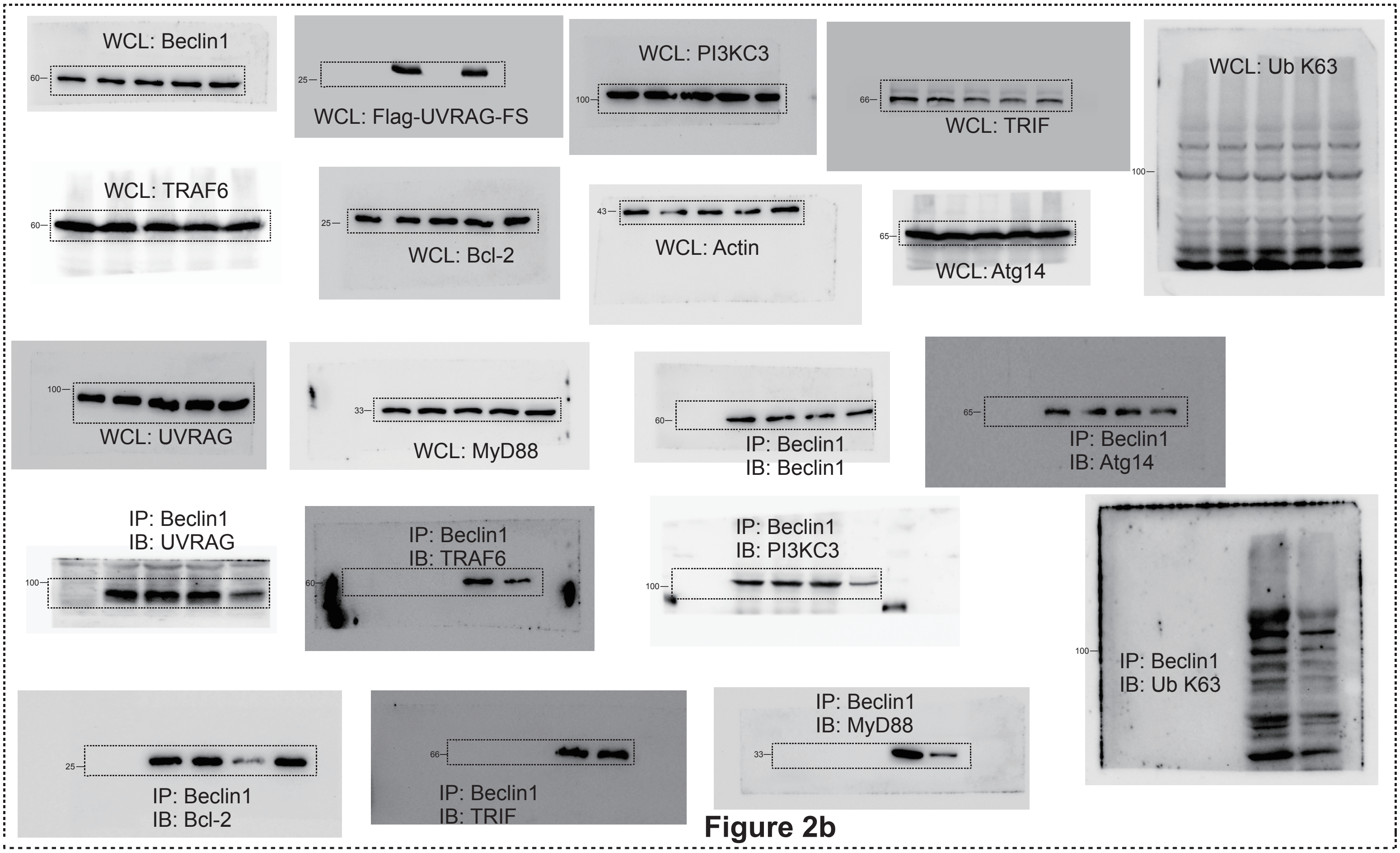

Supplementary Figure 9 : uncropped scans of blots.

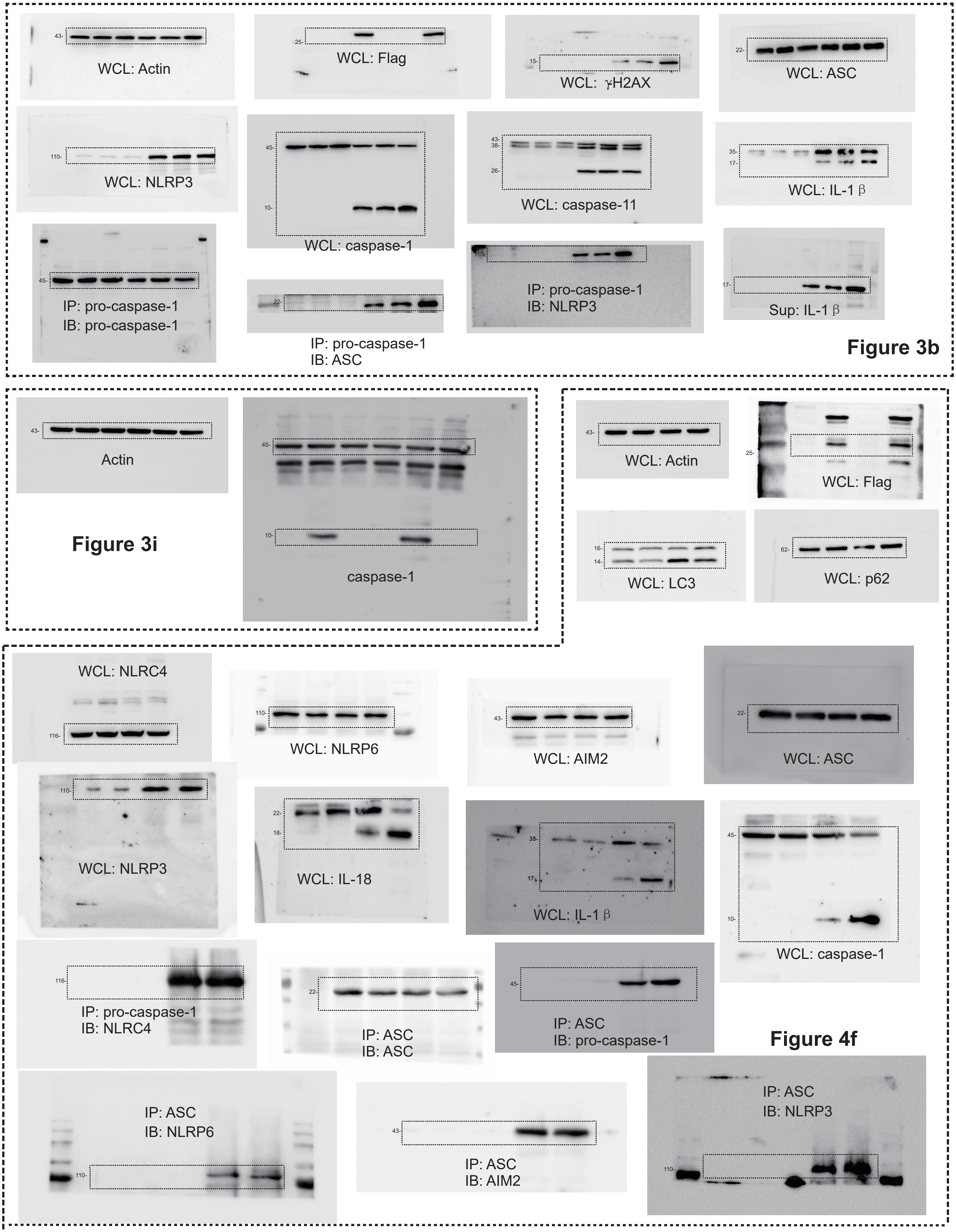

Supplementary Figure 9 (continue)

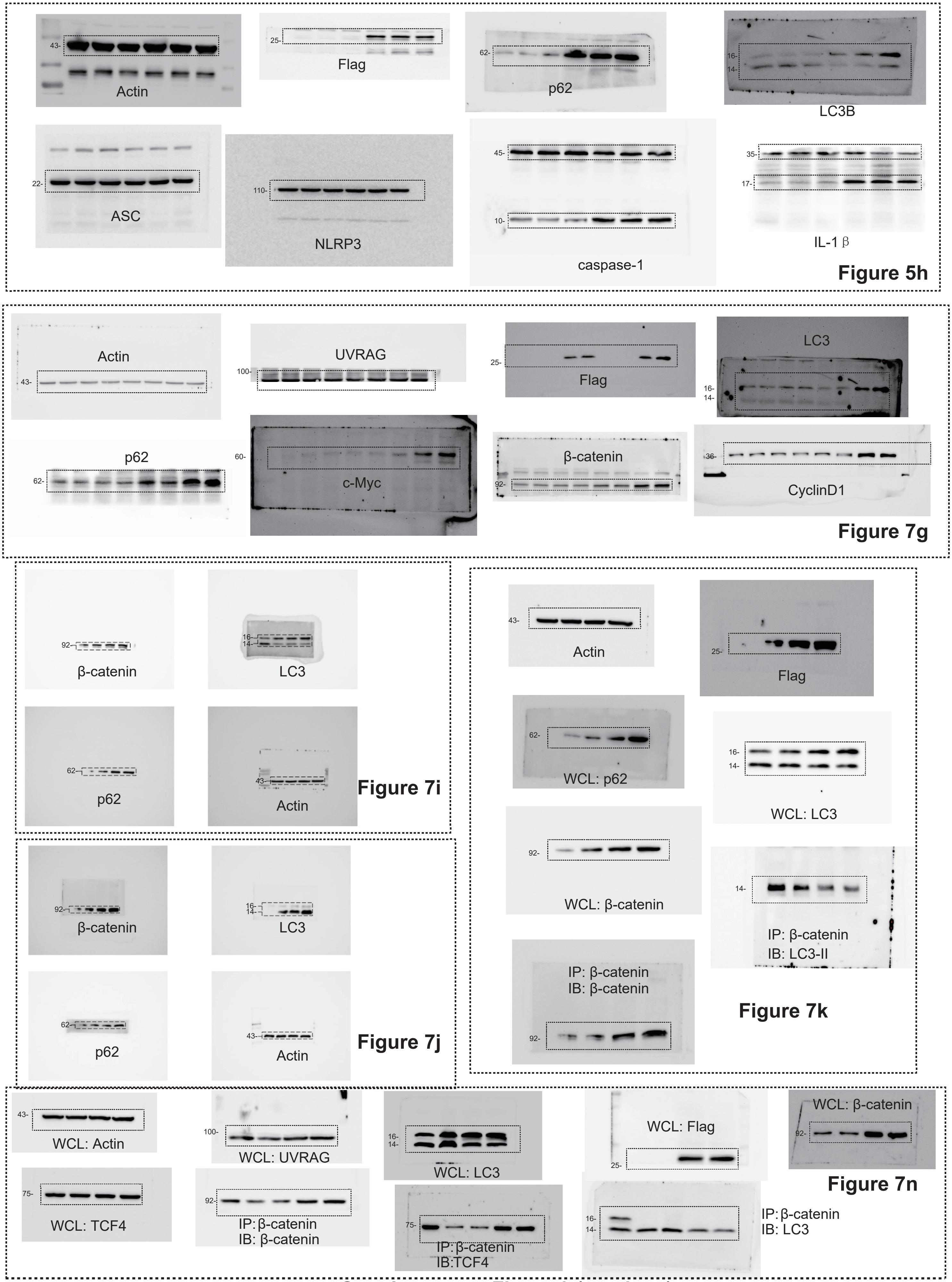

Supplementary Figure 9 (continue)

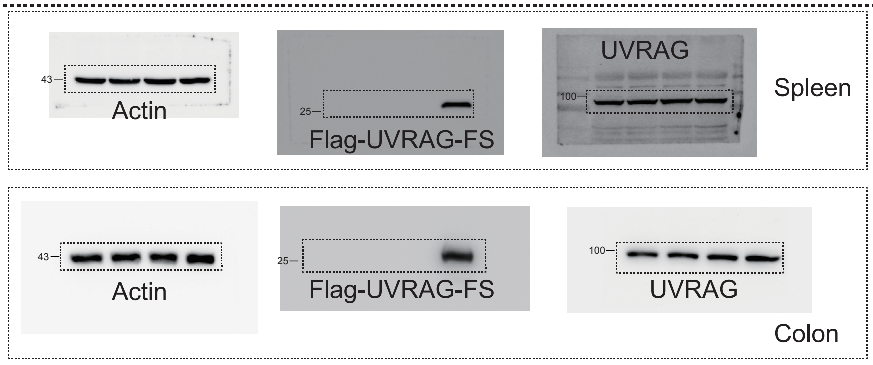

Figure S1c

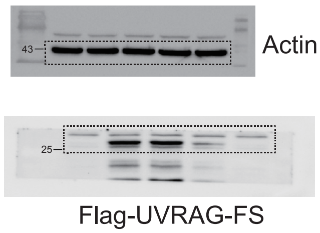

Figure S1g

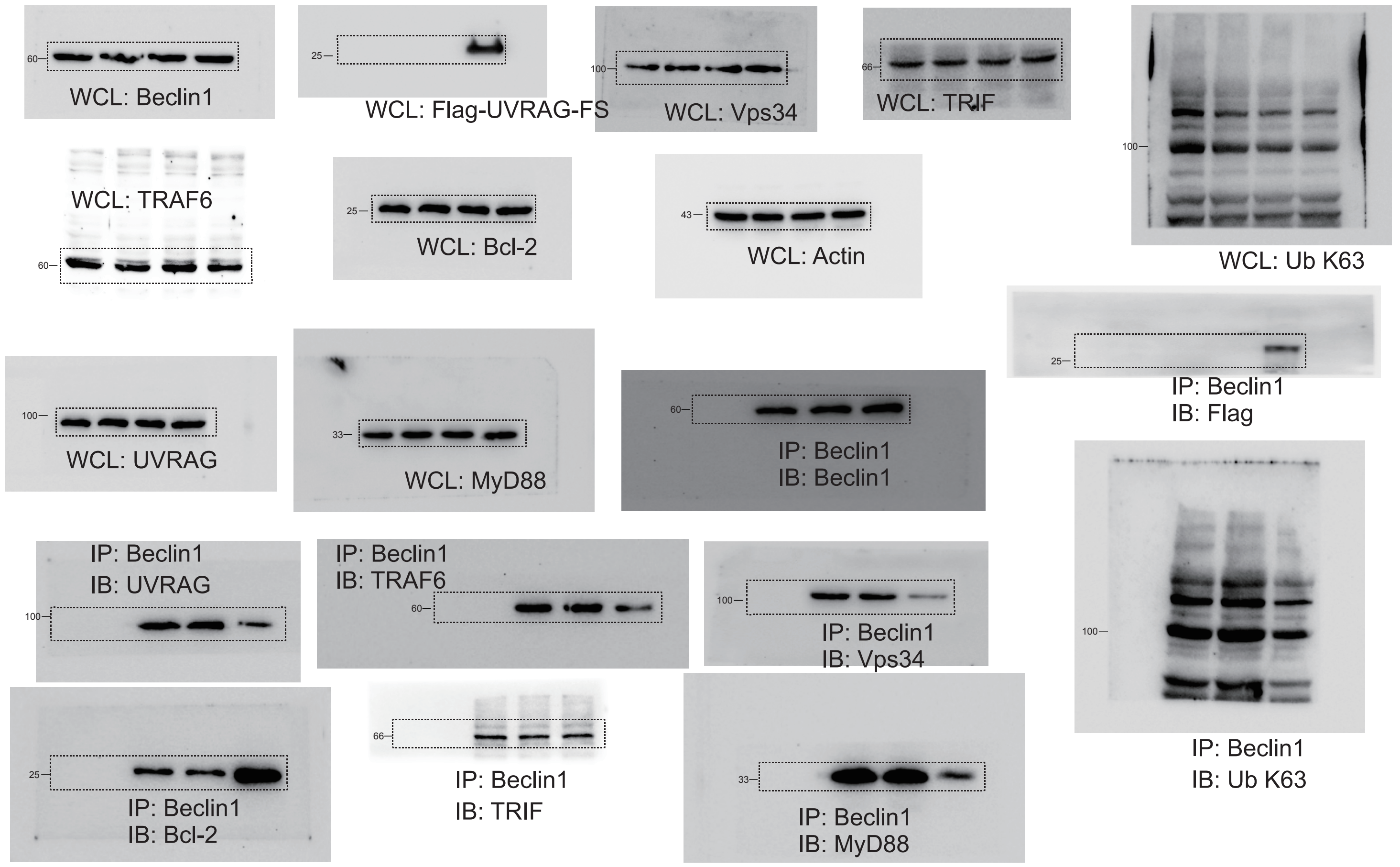

Figure S2b

Supplementary Figure 9 (continue)

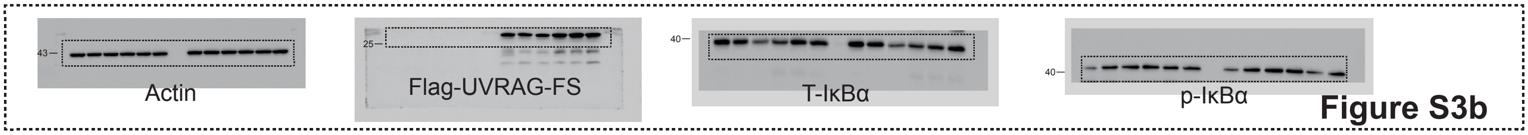

Figure S3b

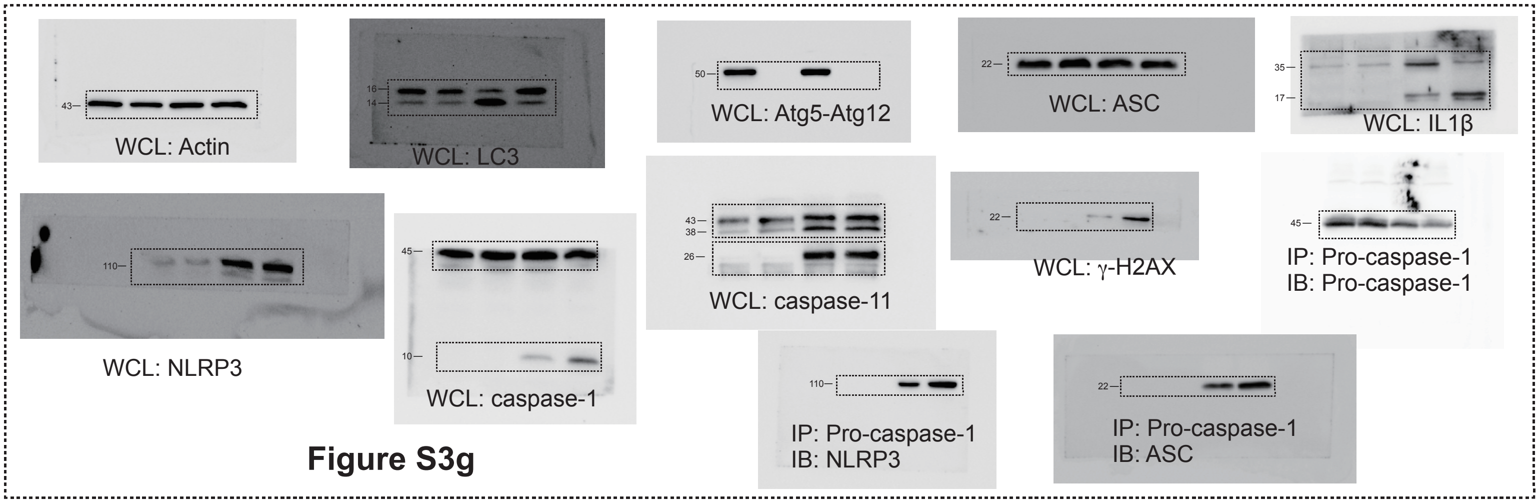

Figure S3g

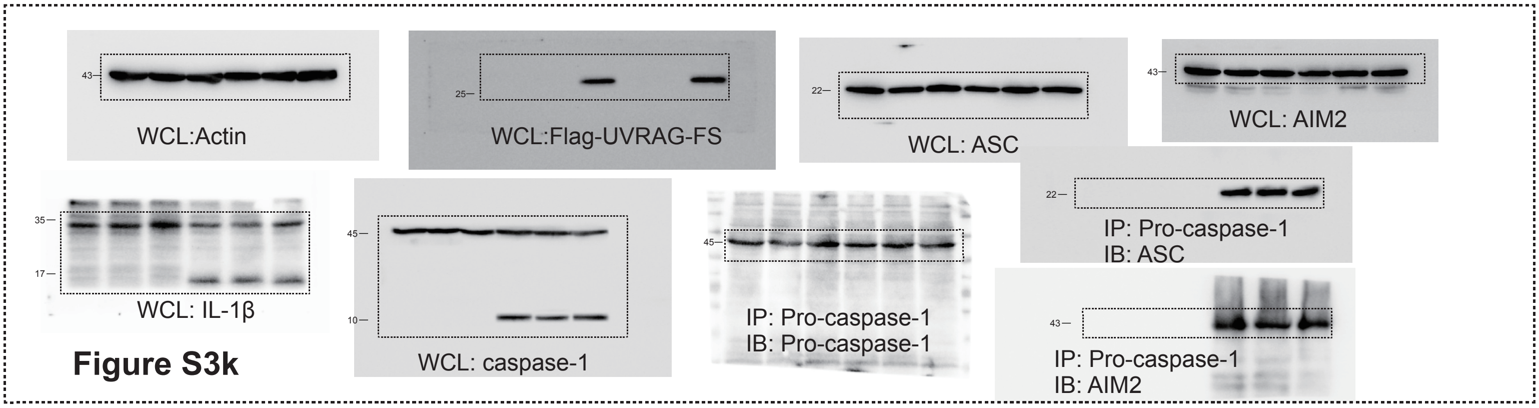

Figure S3k

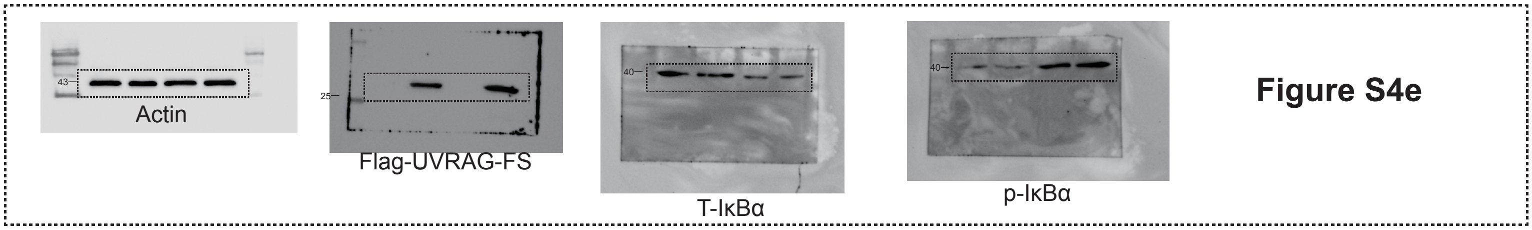

Figure S4e

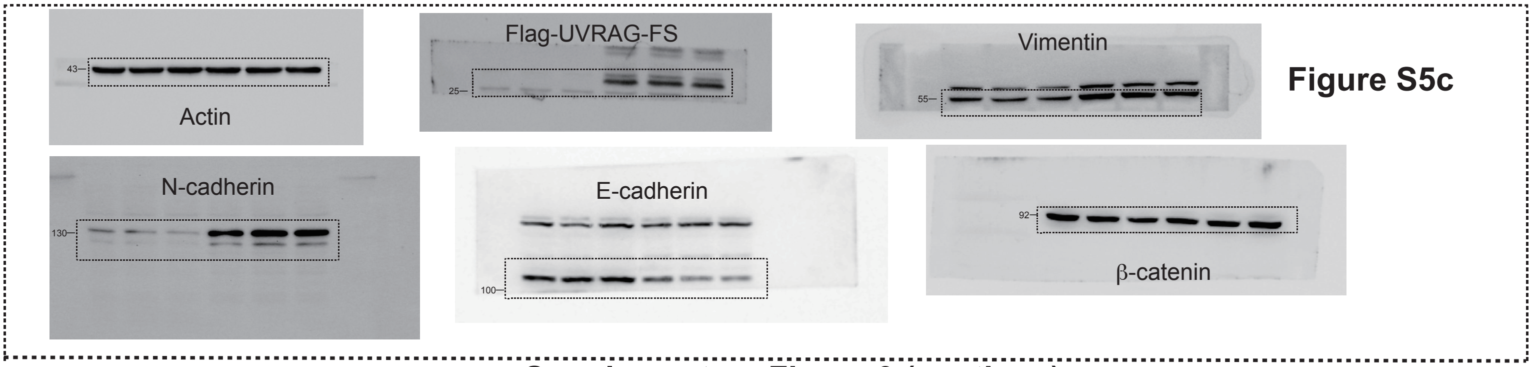

Figure S5c

Supplementary Figure 9 (continue)

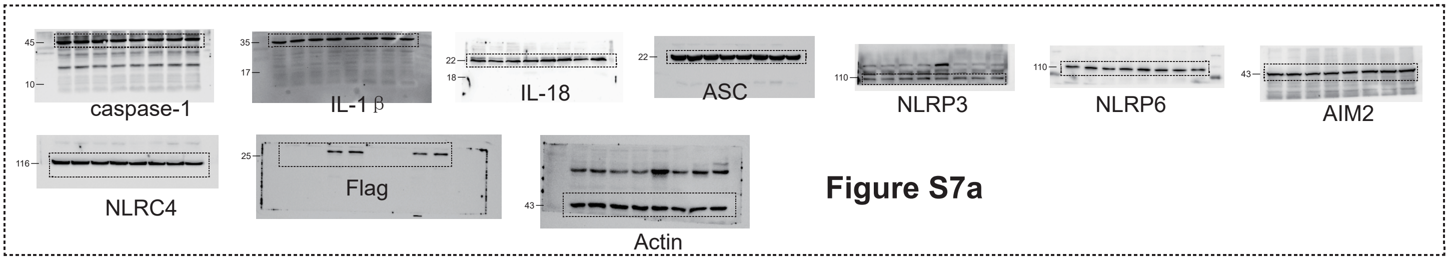

Figure S7a

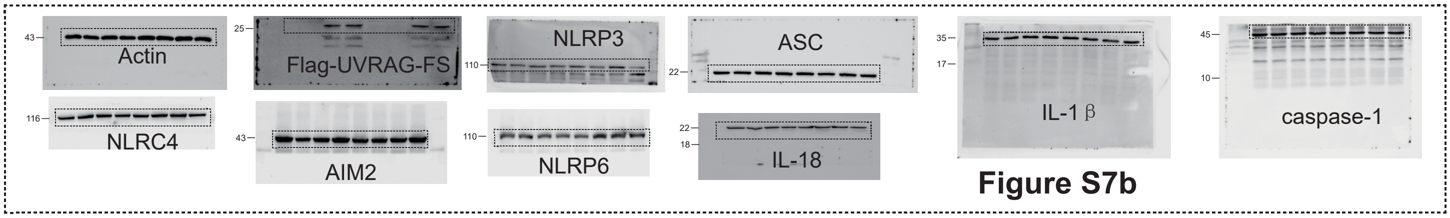

Figure S7b

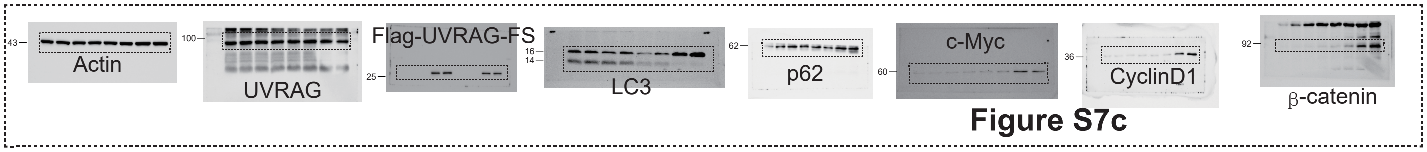

Figure S7c

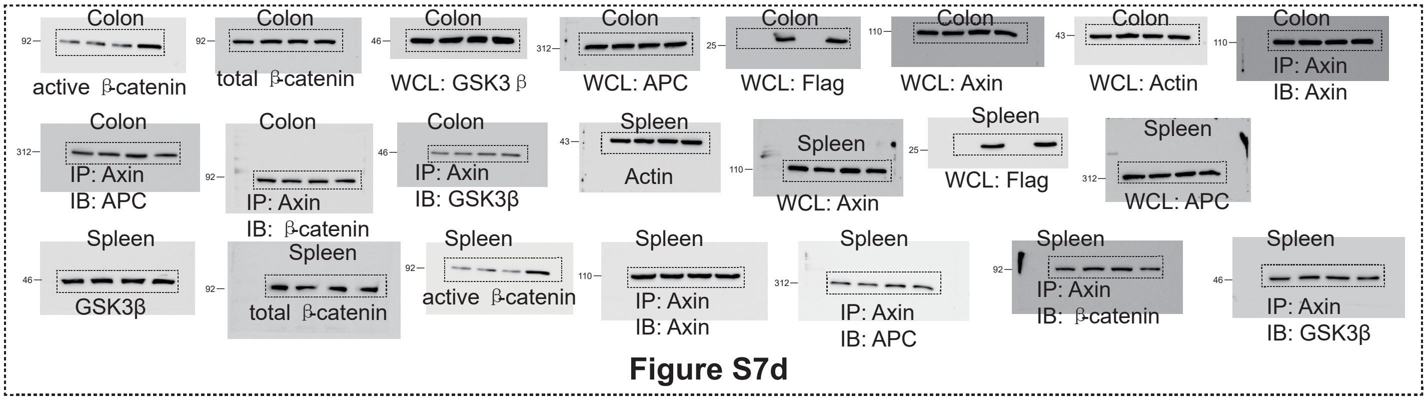

Figure S7d

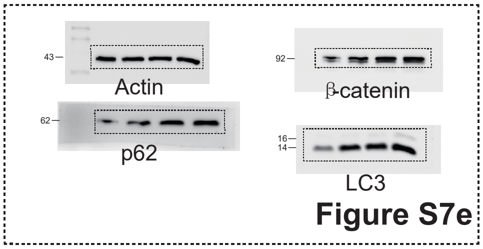

Figure S7e

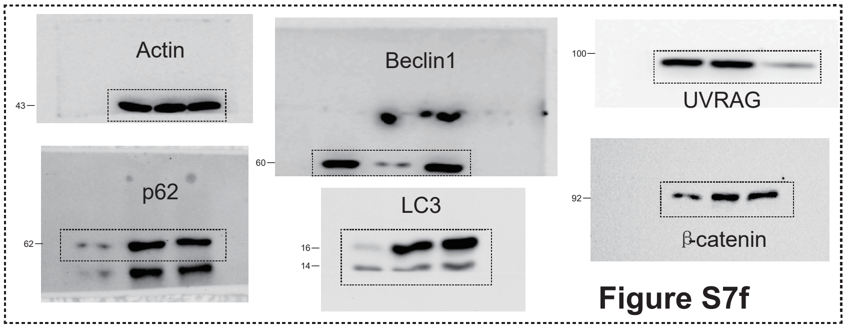

Figure S7f

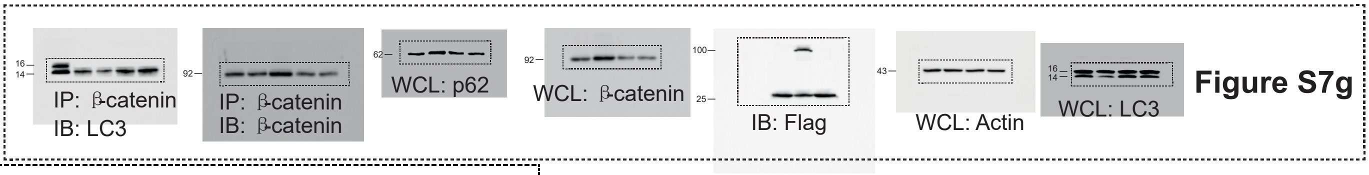

Figure S7g

Figure S7h

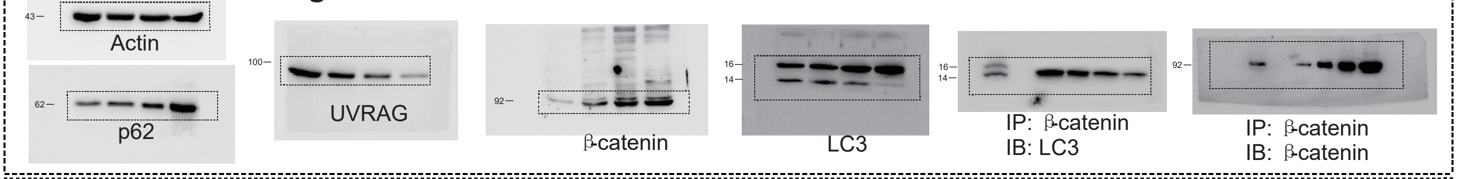

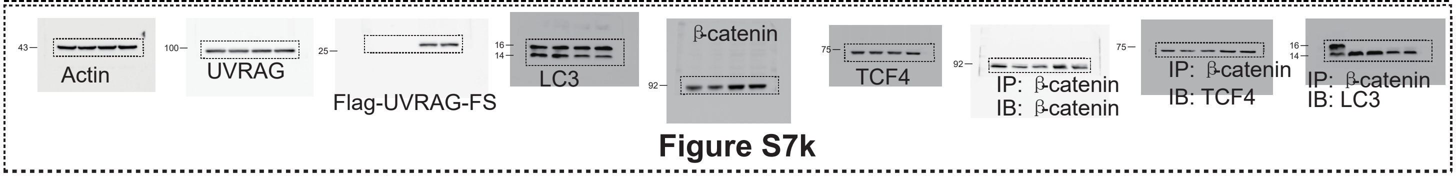

Figure S7k

Supplementary Figure 9 (continue)

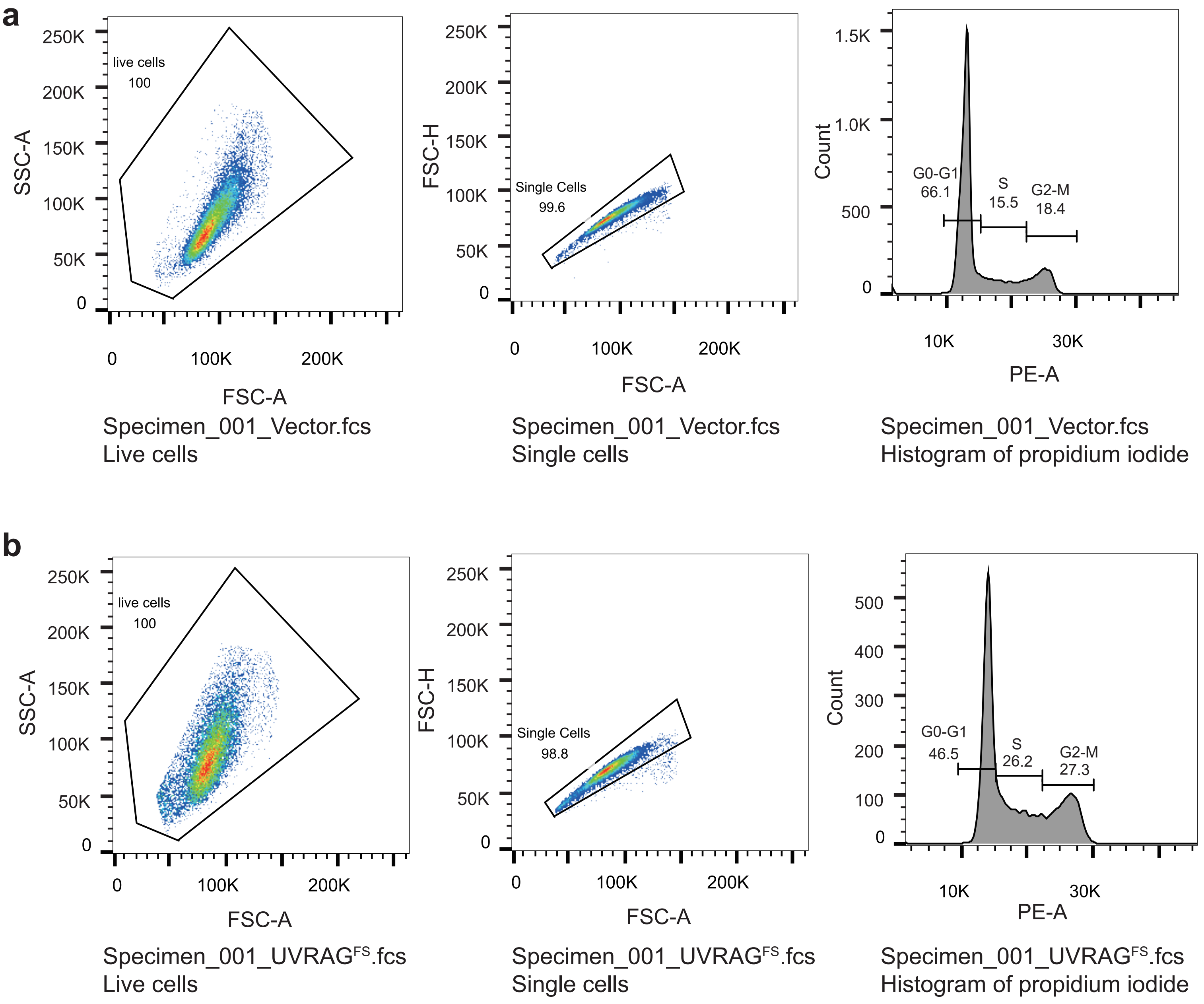

Supplementary Figure 10

Supplementary Figure 10. Gating strategy of flow cytometry for SW480 cells stably expressing vector (**a**) and UVRAG^{FS} (**b**). The cell cycle distribution was assessed with propidium iodide staining and calculated as the percentage of cells containing G0/G1 phase (2n), S phase (between 2n and 4n) and G2/M phase (4n). The results were shown in Figure 7m.

Supplementary Table 1: Primers used for mouse strain genotyping

Gene	Forward (5'-3')	Reverse (5'-3')
rtTA	GGAGCGGGAGAAATGGATATG	AAAGTCGCTCTGAGTTGTTAT<br>GCGAAGAGTTTGTCCTCAACC
TRE-FlagUVRAG^{FS} *#1*	CCCTATCAGTGATAGAGAAC	CAGTACTACACAAGTGAAGT
TRE-Flag-UVRAG^{FS} *#2*	GTGAACCGTCAGATCGCCTG	CAATGTTCCGGGCAGCAATG
Luciferase	TTACAGATGCACATATCGAGG	TAACCCAGTAGATCCAGAGG
GFP-LC3	ATAACTTGCTGGCCTTTCCACT	CGGGCCATTTACCGTAAGTTAT<br>GCAGCTCATTGCTGTTCCTCAA

Supplementary Table 2: Primers used for quantitative PCR

Gene	Forward (5'-3')	Reverse (5'-3')
Flag-UVRAG^{FS}	GGACGACGATGACAAGGG	GTGTAAAGTAGGTATCAAGGAGC
IL-1β	CATCTTTGAAGAAGAGCCCAT	GCCACAGGTATTTTGTCGTT
IL-18	ACACGCTTTACTTTATACCTG	ACTTGGTCATTTATATTCCGTA
IL-6	AGGATACCACTCCCAACAGACC	TGCATCATCGTTGTTCATACA
TNF-α	AGCCGATGGGTTGTACCTTG	ATAGCAAATCGGCTGACGGT
IFN-β	ACTGCCTTTGCCATCCAAGA	CACTGTCTGCTGGTGGAGTT
Actin	GCTGGTCGTCGACAACGGCT	CAAACATGATCTGGGTCATCTTTTC
CXCL1	GCCTATCGCCAATGAG	TTCTGAACCAAGGGAGC
CXCL2	AACCACCAGGCTACAGG	CAGGGTCAAGGCAAACT
CCL2	GAAGGAATGGGTCCAGACAT	ACGGGTCAACTTCACATTCA
CXCL3	TGCCTGAACACCCTACCA	TGGACTTGCCGCTCTT
CCL3	GCAGCCTTTGCTCCCA	TTCCAGGTCAGTGATGTATTCT
CXCL10	CATCCTGCTGGGTCTGA	CGTGGCAATGATCTCAA
COX1	GCCCCAGATATAGCATTCCC	GTTCATCCTGTTCCTGCTCC
c-Myc	GACCTCGACTACGACTCCGT	CGCAACATAGGATGGAGAGCA
Cyclin D1	AAGTGTGACCCGGACTGC	GATGTCCACATCTCGCACG
Axin2	GCAAGTCCAAGCCCCATAGT	GCTTCCTCTAGCTGTGCCAA
18S	TAGAGGGACAAGTGGCGTTC	CGCTGAGCCAGTCAGTGT
